# A Case Report Describing a Rare Presentation of Simultaneous Occurrence of MPO-ANCA-Associated Vasculitis and Rheumatoid Arthritis

**DOI:** 10.1155/2016/9340524

**Published:** 2016-11-06

**Authors:** Nathalie Foray, Tamer Hudali, Muralidhar Papireddy, John Gao

**Affiliations:** ^1^Department of Internal Medicine, Southern Illinois University, 751 North Rutledge, Springfield, IL 62702, USA; ^2^Department of Pathology, Memorial Medical Center, 701 North 1st Street, Springfield, IL 62781, USA

## Abstract

*Background*. Renal-limited myeloperoxidase vasculitis with simultaneous rheumatoid arthritis is reported as a rare occurrence. Review of literature suggests that most patients had a diagnosis of rheumatoid arthritis for several years prior to presenting with renal failure from myeloperoxidase vasculitis.* Case Presentation*. A 58-year-old Caucasian male presented to the hospital experiencing malaise, fevers, decreased oral intake, nausea, and vomiting for one week duration. His past medical history consisted of newly diagnosed but untreated rheumatoid arthritis, hypertension, and non-insulin-dependent diabetes mellitus. He was found to have acute renal failure, proteinuria, and hypoglycemia. Standard therapy, including intravenous fluids, did not improve his acute renal failure. A vasculitis workup resulted in a positive myeloperoxidase anti-neutrophil cytoplasmic antibody (MPO-ANCA). Renal biopsy revealed crescentic glomerulonephritis (GN) pauci-immune type, suggestive of MPO-ANCA-associated vasculitis (MPO-AAV). Treatment consisted of prednisone, cyclophosphamide, and seven cycles of plasmapheresis, in addition to hemodialysis for uremia. Upon discharge, he received hemodialysis for another week and continued treatment with cyclophosphamide and prednisone.* Conclusion*. Patients with longstanding rheumatoid arthritis may develop renal failure due to nonsteroidal anti-inflammatory medication use and AA type amyloidosis; however, necrotizing glomerulonephritis with crescent formation has been rarely reported. This stresses the importance of early recognition and swift initiation of treatment.

## 1. Introduction

One of the rare renal complications of rheumatoid arthritis (RA) is pauci-immune crescentic glomerulonephritis (GN). Due to its rare occurrence and the overlap in clinical features, the diagnosis may be easily missed. The most common causes of renal failure in patients with RA are secondary amyloidosis (AA type) and the use of certain disease modifying drugs, including NSAIDs, gold salts, cyclosporine A, tacrolimus, and D-penicillamine [1, 2]. There are few reported cases of RA that were subsequently diagnosed with pauci-immune necrotizing glomerulonephritis. Messiaen et al. describe two case reports of patients who developed pauci-immune necrotizing glomerulonephritis without vascular manifestations, 9 and 40 years respectively, after the onset of RA [2]. Even glomerulonephritis with RA is quite rare; types such as mesangial proliferative, membranous glomerulonephritis, and even our crescentic glomerulonephritis were usually found in autopsy studies [1]. Previous articles have described patients suffering from acute rheumatic flare that developed deteriorating renal function and were subsequently diagnosed with pauci-immune necrotizing glomerulonephritis [1–3]. Those patients had received treatment for rheumatoid arthritis for years and usually experienced severe forms of RA, such as erosive RA. Typically, patients who develop necrotizing glomerulonephritis have multiorgan involvement with severe rheumatoid arthritis vasculitis [2]. Characteristics of rheumatoid arthritis vasculitis include skin and nail ulcerations/infarcts, rash, and neurologic involvement, in addition to gastrointestinal, cardiac, and ophthalmologic damage [2]. We present a case of simultaneous occurrence of RA and myeloperoxidase positive GN and the effect of timely and accurate diagnosis on the final outcome.

## 2. Case Presentation

A 58-year-old Caucasian male was transferred to our hospital because of renal failure and hypoglycemia. He complained of generalized malaise, subjective fever, decreased oral intake, nausea, vomiting, and diarrhea for 1-week duration. His medical history is significant for RA diagnosed by his primary care physician, which was not in an acute flare. Through his PCPs office he was found to have a positive homogenous ANA, although a negative rheumatoid factor at the time. He had complained of pain, stiffness, and occasional swelling of elbow and hand joints, but no X-rays were obtained at that time. He was sent for a referral for rheumatology but cancelled two of his appointments by the time he was hospitalized. Based on the 2010 American College of Rheumatology/European League Against Rheumatism Collaborative Initiative Rheumatoid Arthritis Classification, he would receive 5 points for his joint symptoms (>10 joints affected with at least one small joint) and 1 point based on the duration of symptoms (>6 weeks), classified as having rheumatoid arthritis (≥6/10 points) [4]. He also has hypertension and diabetes mellitus type II. His home medications are lisinopril, metformin, and glipizide. He denied use of over the counter medications including nonsteroidal anti-inflammatory medications (NSAIDs). On physical exam he appeared euvolemic and afebrile, with a blood pressure of 126/72 mmHg. The rest of his physical examination was unremarkable, aside from hands, elbows, knees, and ankles that were stiff/sore variably, but there was no synovitis, warmth, or rash. His labs from the outlying hospital showed a creatinine of 6.6 mg/dL (his baseline creatinine is 0.8 mg/dL) and blood glucose of 30 mg/dL.

**Figure 1:**
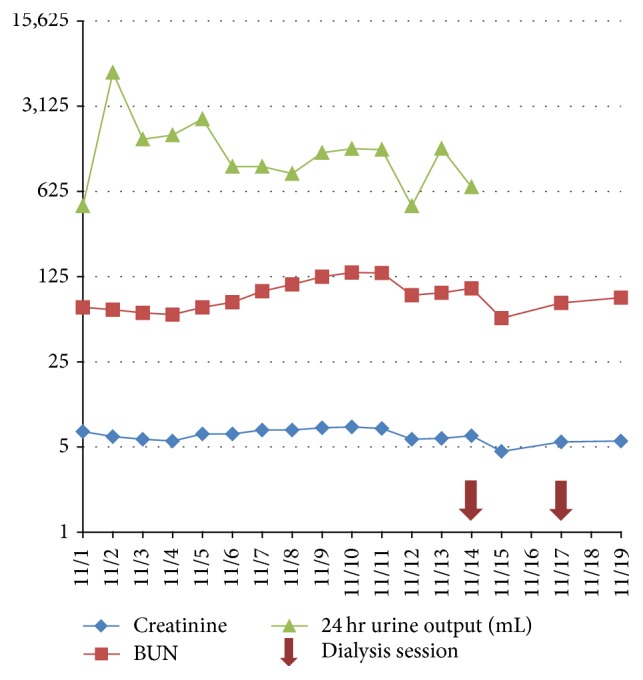
Creatinine and BUN trend during hospitalization. The large arrows reflect the days he had hemodialysis.

**Figure 2:**
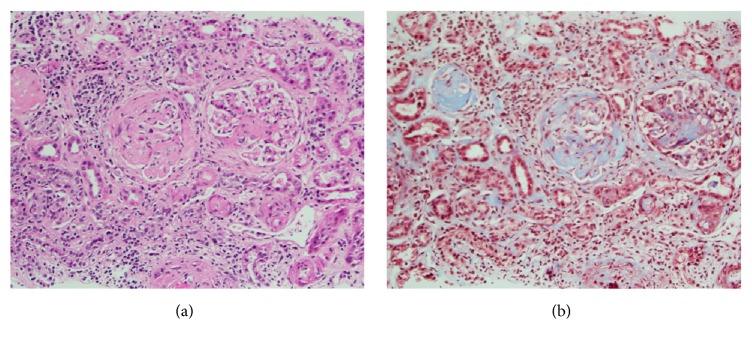
Renal biopsy revealing crescentic glomerulonephritis (GN) pauci-immune type suggestive of MPO. H&E stain and trichrome stain.

Primary workup for the acute kidney injury included a creatinine and blood urea nitrogen (BUN) trend as shown in Figure 1 (initial creatinine of 6.1 mg/dL and BUN of 67 mg/dL). After correcting his hypoglycemia, we started IV fluids for the acute kidney injury (AKI). Fractional excretion of sodium at admission was 4.5%. Other urine studies showed microscopic hematuria and nephritic range proteinuria with a random protein : creatinine ratio of 1.4 and negative for urine eosinophils. Complete blood count on admission revealed a leukocytosis of 11 K/cuMM, hemoglobin was 8.3 gm/dL, and he had a thrombocytosis of 422 K/cuMM. Erythrocyte sedimentation rate was not obtained until the 12th day of hospitalization and results were normal at 2 mm/hr. CRP was not obtained. C3 complement levels were low-normal at 72 mg/dL and C4 complement was 18 mg/dL. Results of protein electrophoresis were normal. Results for anti-streptolysin O antibody and hepatitis profile were negative. Vasculitis workup was remarkable for a positive p-antineutrophil cytoplasmic antibody (p-ANCA) with a titer of 1 : 640, positive myeloperoxidase- (MPO-) ANCA of >8.8 antibody index, and positive antinuclear antibody with a titer of 1 : 80. Results were negative for anti-glomerular basement membrane antibodies and anti-proteinase-3 antibody. Rheumatoid factor and anti-CCP were not tested during this hospitalization. Renal biopsy on hospital day 5 showed crescentic glomerulonephritis (GN) pauci-immune type suggestive of MPO-AAV as shown in Figures 2(a) and 2(b).

The biopsy in Figures 2(a) and 2(b) shows sclerotic glomeruli with focal fibrinoid necrosis and cellular crescent formation. The nonsclerotic glomeruli range from normocellular to hypercellular and contain a variable mesangial matrix prominence. Mild to moderate interstitial inflammation consisting of lymphocytes and plasma cells is present. The trichrome stain highlights mild to moderate interstitial fibrosis and the sclerotic glomeruli (H&E, trichrome stains 200x magnification).

In further detail, there are eight H&E, two PAS, two trichrome, and three Jones stains. The biopsy consists of a single core fragment of renal tissue containing cortex and is adequate for evaluation. On one representative level of the biopsy, six total glomeruli are identified, one of which is sclerotic. On the recut deeper levels, approximately two glomeruli are involved by cellular crescent formation. A third glomerulus, which is partially represented, is suspicious for cellular crescent formation. One glomerulus contains an area of fibrinoid necrosis. The nonsclerotic glomeruli range from normocellular to hypercellular and contain a variable mesangial matrix prominence. One glomerulus contains a segmental scar and some glomeruli contain rare neutrophils.

The Jones stain highlights foci of glomerular basement membrane breakage and variable wrinkling. The PAS stain highlights moderate tubular atrophy and moderate tubulitis. Some tubular lumens contain debris and red blood cells. The trichrome stain highlights mild to moderate interstitial fibrosis. Mild to moderate interstitial inflammation consisting of lymphocytes and plasma cells is noted. The tissue does not contain small arteries. Arterioles are narrowed with mild hyalinization noted.

Immunofluorescence microscopy is performed on the renal tissue containing eight total glomeruli with two to three glomeruli being sclerotic. Weak granular mesangial and paramesangial staining is identified with the IgM. 2+granular mesangial staining is noted with C3. Some positive fibrinogen staining is identified in Bowman's space of some glomeruli. No significant glomerular staining is noted with IgG, IgA, C1q, kappa, or lambda. Casts stain with albumin, IgG, IgA, IgM, C3, kappa, and lambda. A few arterioles stain with IgM and C3.

Examination of five electron microscope thick sections prepared from the tissue submitted for electron microscopy reveal the presence of six total glomeruli, five of which are sclerotic. The ultrastructural evaluation is performed on thick section #2 containing one nonsclerotic glomerulus which appears to be involved by crescent formation and has significant damage. The ultrastructural evaluation reveals focal glomerular basement membrane breakage. Glomerular basement membranes appear variable in width and wrinkled in areas. There is no overt evidence of electron dense immune deposits associated with the glomerular basement membranes. Patchy foot process effacement is identified and the mesangium is variably expanded and contains sclerosis in areas. There is no overt evidence of electron dense immune deposits within the mesangial area of the glomerulus. Some fibrin deposition is noted associated with the glomerulus.

Because the patient presented with decreased oral intake and GI fluid loss, we considered a prerenal etiology. This diagnosis was quickly ruled out based on the urine studies and the lack of response with intravenous fluids. The differential diagnosis for an AKI associated with nephritic range proteinuria and positive vasculitic parameters as mentioned previously was glomerulonephritis secondary to microscopic polyangiitis or polyangiitis with granulomatosis, focal segmental GN, and amyloidosis. Kidney biopsy showing a pauci-immune crescentic GN confirmed the diagnosis.

We started the patient on methylprednisolone 1 gram IV on hospital day 5, after discovering that he had crepitations, a new bat-wing appearance on chest X-ray, and hemoptysis. He was transitioned to oral prednisone 80 mg/day and cyclophosphamide 150 mg/day on hospital day 6 and started on prophylactic trimethoprim-sulfamethoxazole 80–400 mg every Monday, Wednesday, and Friday for* Pneumocystis jiroveci* pneumonia. In addition, he received 7 rounds of plasma exchange for pulmonary hemorrhage. His BUN steadily increased, which was suspected to be secondary to his steroid therapy. He developed worsening hyperkalemia and a persistent elevation in BUN without signs of recovery; thus, hemodialysis was initiated on hospital day 11. During his stay, he was transfused with packed red blood cells and given an injection of epoetin alfa on hemodialysis days. He had two cycles of hemodialysis during his hospitalization.

The pain and joint stiffness in the patient's hands improved significantly with treatment and he was able to resume playing the guitar. After discharge from the hospital, the patient continued on prednisone therapy (80 mg/day) for 2–4 weeks with a taper over 5 months and he continued with cyclophosphamide 150 mg/day for a total of 3–6 months. Following discharge, his renal functions gradually improved and hemodialysis was discontinued a week later. He was sent a laboratory order for anti-CCP but was unable to complete the test secondary to out-of-pocket costs. Our patient has been followed up with monthly CBC and CMP and has been following up with nephrology every three months since his discharge in 2014. Cyclophosphamide was discontinued in February 2016, and prednisone was tapered down and discontinued by July 2016. His ANCA markers, Proteinase 3 antibody and myeloperoxidase antibody, were tested in May 2016. Proteinase 3 antibody was 3 U/mL (normal < 15 U/mL) and myeloperoxidase antibody was 4 U/mL (normal < 15 U/mL). He does have advanced CKD stage 4-5, as his renal function has slowly worsened as years passed, with his creatinine in May 2016 5.3 mg/dL, increasing to 6.2 mg/dL September 2016. He has not been started on hemodialysis yet; however, it is assumed that the increasing levels of creatinine signify he will need hemodialysis in the near future.

## 3. Discussion

Previous case reports describe MPO-ANCA-associated crescentic glomerulonephritis in adults between the ages of 20 and 50 [5]. Although the mechanism of action is unknown, a study by Kurita et al. speculated that there is a decrease in renal function after the onset of microhematuria. Although the patients studied had incremental increases in creatinine, their renal biopsies revealed advanced renal disease, with glomerular sclerosis and less crescent formation. It is also hypothesized that treatment with immunosuppressants, such as methotrexate and infliximab, can influence crescentic glomerulonephritis activity [5]. Our patient, however, did not receive drug therapy before presenting to the hospital.

Our patient did not have symptoms characteristic of rheumatoid arthritis vasculitis. Because RA varies widely from patient to patient, it is important to find markers that could indicate disease progression. It has been found that 12% of patients with RA with varying disease activity levels were positive for anti-MPO antibodies [6–8]. None of the patients studied had systemic vasculitis. High titers of rheumatoid factor correlate with early bone erosion, and individuals with high-risk alleles such as HLA DRB1 have a greater risk of disease progression [9]. The incidence of ANCA in RA is as high as 40%, and individuals have expressed p- and c-ANCA staining patterns [7]. In a study by Braun et al., 16% of the patients had a positive ANCA and all showed a perinuclear pattern (p-ANCA). RA patients with p-ANCA experience greater disease severity, have higher inflammatory markers, and develop vascular and pulmonary complications. Another study showed that 5 out of 61 patients who were p-ANCA positive had pulmonary involvement [7]. Our patient, who is p-ANCA positive, developed pulmonary hemorrhage.

We started our patient on high-dose methylprednisolone on hospital day 5 for hemoptysis and pulmonary involvement and transitioned him to oral prednisone and cyclophosphamide on hospital day 6. Kurita et al. classified RA patients with MPO-ANCA based on RA treatments prior to presentation, therapies provided, and the patient's outcome [5]. They described three patients with renal manifestations who improved with therapy consisting of a combination of prednisolone and cyclophosphamide with or without methylprednisolone. One patient, who did not receive therapy after diagnosis, developed end-stage renal disease [5]. Patients with primary manifestations of hemoptysis and significant proteinuria either died or developed end-stage renal failure, despite receiving therapy [5].

We present the case of a patient with a recent diagnosis of rheumatoid arthritis who developed rapid deterioration of renal function that was not caused by disease modifying medications. After a full renal workup the patient was found to have pauci-immune necrotizing glomerulonephritis. Thus, it is important to obtain a full renal workup in individuals with rheumatoid arthritis when creatinine levels are elevated at baseline and do not improve despite typical interventions. Early recognition followed by adequate treatment could prevent or slow the progression of renal failure to end-stage renal disease.

## 4. Conclusion

It is important to understand that swift rises in creatinine can occur in individuals with acute kidney injury or acute tubular necrosis if there is hemodynamic instability; however, it is important to consider other causes of persistently rising creatinine levels despite typical interventions. We recommend a full renal workup on patients whose creatinine levels continue to rise despite typical interventions, such as eliminating offending agents, providing hydration, and maintaining hemodynamics. The workup should include serum and urine protein electrophoresis, measurements of complement levels, serologies, and, if still inconclusive, renal biopsy. It is important to note that patients may develop complications of pauci-immune crescentic glomerulonephritis, such as pulmonary hemorrhage. If physical examination demonstrates change or if the patient develops hemoptysis, further workup and treatment with plasma exchange is indicated. Individuals with MPO-AAV will require long-term treatment with cyclophosphamide, steroids, and prophylactic medications against* Pneumocystis jiroveci* pneumonia.

## Abbreviations

MPO: Myeloperoxidase
ANCA: Anti-neutrophil cytoplasmic antibody
GN: Glomerulonephritis
RA: Rheumatoid arthritis
DMARDs: Disease modifying antirheumatic drugs
NSAIDs: Nonsteroidal anti-inflammatory medications
mmHg: Millimeters of mercury
mg: Milligram
dL: Deciliter
BUN: Blood urea nitrogen
AKI: Acute kidney injury
K/cuMM: Thousand per cubic millimeter
mm/hr: Millimeter per hour
p-ANCA: p-Anti-neutrophil cytoplasmic antibody
anti-CCP: Anti-cyclic citrullinated peptide antibody
H&E: Hematoxylin and eosin stain
mg: Milligram
RA: Rheumatoid arthritis
c-ANCA: c-Anti-neutrophil cytoplasmic antibody
AAV: ANCA-associated-vasculitis.

## References

[1] Horak P., Smrzova A., Krejci K., Tichy T., Zadrazil J., Skacelova M. (2013). Renal manifestations of rheumatic diseases. A review. *Biomedical Papers of the Medical Faculty of the University Palacky, Olomouc, Czechoslovakia*.

[2] Messiaen T., M'bappé P., Boffa J. J. (1998). MPO-ANCA necrotizing glomerulonephritis related to rheumatoid arthritis. *American Journal of Kidney Diseases*.

[3] Qarni M. U., Kohan D. E. (2000). Pauci-immune necrotizing glomerulonephritis complicating rheumatoid arthritis. *Clinical Nephrology*.

[4] Aletaha D., Neogi T., Silman A. J. (2010). 2010 Rheumatoid arthritis classification criteria: an American College of Rheumatology/European League Against Rheumatism collaborative initiative. *Annals of the Rheumatic Diseases*.

[5] Kurita N., Mise N., Fujii A. (2010). Myeloperoxidase-antineutrophil cytoplasmic antibody-associated crescentic glomerulonephritis with rheumatoid arthritis: a comparison of patients without rheumatoid arthritis. *Clinical and Experimental Nephrology*.

[6] Cambridge G., Williams M., Leaker B., Corbett M., Smith C. R. (1994). Anti-myeloperoxidase antibodies in patients with rheumatoid arthritis: prevalence, clinical correlates, and IgG subclass. *Annals of the Rheumatic Diseases*.

[7] Braun M. G., Csernok E., Schmitt W. H., Gross W. L. (1996). Incidence, target antigens, and clinical implications of antineutrophil cytoplasmic antibodies in rheumatoid arthritis. *Journal of Rheumatology*.

[8] Coremans I. E. M., Hagen E. C., Daha M. R. (1992). Antilactoferrin antibodies in patients with rheumatoid arthritis are associated with vasculitis. *Arthritis & Rheumatism*.

[9] Vittecoq O., Jouen-Beades F., Krzanowska K. (2000). Prospective evaluation of the frequency and clinical significance of antineutrophil cytoplasmic and anticardiolipin antibodies in community cases of patients with rheumatoid arthritis. *Rheumatology*.

